# Molecular mapping and identification of quantitative trait loci for domestication traits in the field cress (*Lepidium campestre* L.) genome

**DOI:** 10.1038/s41437-020-0296-x

**Published:** 2020-02-19

**Authors:** Zeratsion Abera Desta, Dirk-Jan de Koning, Rodomiro Ortiz

**Affiliations:** 1 Department of Plant Breeding, Swedish University of Agricultural Sciences, Sundesvagen 10 Box 101, 23053 Alnarp, Sweden; 2 Department of Animal Breeding and Genetics, Swedish University of Agricultural Sciences, Box 7023, 75007 Uppsala, Sweden

**Keywords:** Genetic linkage study, Plant breeding, Quantitative trait loci, Plant domestication

## Abstract

*Lepidium campestre* (L.) or field cress is a multifaceted oilseed plant, which is not yet domesticated. Moreover, the molecular and genetic mechanisms underlying the domestication traits of field cress remain largely elusive. The overarching goal of this study is to identify quantitative trait loci (QTL) that are fundamental for domestication of field cress. Mapping and dissecting quantitative trait variation may provide important insights into genomic trajectories underlying field cress domestication. We used 7624 single nucleotide polymorphism (SNP) markers for QTL mapping in 428 F_2_ interspecific hybrid individuals, while field phenotyping was conducted in F_2:3_ segregating families. We applied multiple QTL mapping algorithms to detect and estimate the QTL effects for seven important domestication traits of field cress. Verification of pod shattering across sites revealed that the non-shattering lines declined drastically whereas the shattering lines increased sharply, possibly due to inbreeding followed by selection events. In total, 1461 of the 7624 SNP loci were mapped to eight linkage groups (LGs), spanning 571.9 cM map length. We identified 27 QTL across all LGs of field cress genome, which captured medium to high heritability, implying that genomics-assisted selection could deliver domesticated lines in field cress breeding. The use of high throughput genotyping can accelerate the process of domestication in novel crop species. This is the first QTL mapping analysis in the field cress genome that may lay a foundational framework for positional or functional QTL cloning, introgression as well as genomics-assisted breeding in field cress domestication.

## Introduction

A biennial self-pollinated plant, with relatively small genome size (2*n* = 2*x* = 16 and approximately 203.5 *Mb*) (Desta et al. 2019), field cress (*Lepidium campestre* L.) belongs to the Brassicaceae family. In addition to being a potential oilseed plant (Merker and Nilsson 1995; Nilsson et al. 1998), the winter hardy field cress can be: (i) undersown with a cereal as a catch crop (Merker et al. 2010) to recycle vulnerable nutrients (e.g. nitrogen and phosphorous) from leaching (Ulén and Aronsson 2018); (ii) utilized as an eco-friendly solution to safeguard the environment and alleviate the negative consequences of climate change, such as nitrogen pollution and eutrophication; and (iii) used to domesticate new crops as a viable option to feed an ever-increasing global population, thus addressing the future food-security concerns.

Despite its potential uses, field cress remains undomesticated due to lack of research on the molecular and genetic mechanisms controlling its domestication traits. As majority of these traits are quantitative in nature (Doebley et al. 2006), mapping and analysis of quantitative trait loci (QTL) is the key starting point.

Crop domestication is a dynamic process that results into morphologically and physiologically divergent crops compared with their wild progenitors. Historically, domestication syndrome was done considering the properties of the wild variant of a crop (Hammer 1984), such as reduced seed shattering, free-threshing, large seeds or reduced seed dormancy, to obtain an edible variant of that crop species with various modified traits. Thus, crop domestication is a process of altering the morphological or physiological traits of underlying genes to achieve the intended end usage (Doebley et al. 2006). In addition to food and technological innovation, domestication can also be used for genome evolution and adaptive selection (Purugganan and Fuller 2009; Walsh and Lynch 2018).

Similar to other members of Brassicaceae (Gan et al. 2008, 2016; Jie et al. 2017), pod dehiscence (release of seeds from the mother plant) is a domestication trait resulting in yield loss in field cress cultivation. Many experimental studies have isolated and characterized the genes associated with seed shattering, e.g., in barley (Pourkheirandish et al. 2015), wheat (Simons et al. 2006), rice (Li et al. 2006), sorghum (Lin et al. 2012), and tomato (Vrebalov et al. 2009). Therefore, cloning of genes attributed to pod shattering QTL is essential to accelerate the process of domestication and improve the yield.

Field cress has significant variations in traits, such as leaf morphology (e.g. leaf indentation) and plant architecture. However, these patterns (e.g. leaf shape, leaf arrangement) are not random but highly organized and systematically controlled variations (Juenger et al. 2005; Klingenberg 2002). The *teosinte branch 1* (*tb1*) (Doebley et al. 1995; Doebley et al. 1997) and *barren stalk 1* (*ba1*) in maize (Gallavotti et al. 2004) and *PROSTRATE GROWTH 1* (*PROG1*) in rice (Jin et al. 2008) are some of the well-defined genes controlling the architecture of plants. Thus, an extensive research on morphology-related traits of field cress will not only accelerate the domestication process, but also contribute to the understanding of plant growth and development across the members of Brassicaceae.

Due to their frequent and stable occurrence across the genomes, high-throughput sequencing platforms of single nucleotide polymorphism (SNP) discoveries have gained much attention and popularity (Brookes 1999; Shastry 2002). Notably, both the availability and distribution of SNPs across chromosomes lead to the establishment of ultrahigh density linkage maps (Vukosavljev et al. 2016). Linkage analysis can be used to determine how the patterns of parental genes paired with the marker loci (e.g. SNP loci) are co-inherited in the derived offspring population.

This study aims to identify the putative QTL regions pivotal in field cress domestication. It is the first QTL analysis for domestication traits using 7624 SNP markers in 428 F_2_ population of *L. campestre*. We identified major and minor QTL, whose functional validation may not only provide a venue to understand the process of domestication, but also illuminate the positional or functional QTL cloning, introgression of desirable QTL, as well as genomics-assisted breeding strategies in field cress domestication.

## Materials and methods

### Plant material and phenotyping

Two half-sib F_1_ plants were obtained by crossing two separate field cresses as maternal parents with *L. heterophyllum* as a common pollen donor parent. A total of 428 F_2_ progenies were obtained by selfing the F_1_ plants. Although field cress is biennial and *L*. *heterophyllum* is a perennial species, both plants are self-pollinating type. Field phenotyping was performed in the derived individuals of F_2:3_ families to accommodate the trial of segregating populations with replications.

**Fig. 1:**
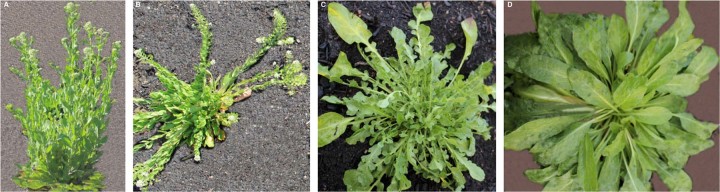
Domestication traits of *Lepidium*. **a** Erect (up-right) type of plant architecture. **b** Prostrate type of plant architecture. **c** Indented (lobed) leaf morphology. **d** Non-indented (non-lobed) leaf morphology. Photo credit by Desta.

Seedlings were initially raised in a greenhouse and vernalized in a cold room for about six weeks. In 2014, the F_2:3_ individual lines were planted in field using three blocks with six replications. We recorded seven important agro-morphological traits for domestication and yield improvement: plant height (cm), inflorescence height (cm), stem number, plant architecture, pod shattering, growth habit, and leaf morphology. Plant architecture was recorded based on the angle of the stem positions, i.e., erect plant architecture is upright position of stems (Fig. 1a), whereas the semi-erect plant architecture is an angle lower than erect but higher than semi-prostrate position. Prostrate position is the stems lying on the ground (Fig. 1b), while semi-prostrate is an angle positioned between semi-erect and prostrate architectures.

Leaf morphology was determined based on the extent of indentation or lobing, e.g., highly indented, medium indented, and non-indented leaves (see Fig. 1c, d). Plants were left in the field for an extended period of time (in 2015) to investigate the perenniality, and those which eventually survived till the next season were considered perennial. Pod shattering was evaluated as shattering (for sensitive genotype) and non-shattering (for reduced shattering) genotypes. Individuals retaining approximately <75% and ≥75% seeds in the mother plants were recorded as shattering and non-shattering genotypes, respectively. To verify this, we further evaluated the F_4_ progenies in 2015 at Alnarp (southern Sweden). Next, the selected individuals of F_4_ progenies were again phenotyped with F_5_ progenies in 2016 both at Alnarp and Uppsala (central Sweden).

### Phenotypic analysis

Using the mixed models, variance components were estimated and significance tests were determined through log-likelihood ratio. To analyze the variance generated from the mixed models, we used the following matrix notation:

$$Y = {\boldsymbol{X}}{\boldsymbol{\beta}} + {\boldsymbol{Zu}}\, + \in ,$$

where *Y* is a known vector of phenotypic trait observations; ***X*** is the known design matrix of fixed predictors, relating observations in *Y* to ***β***; ***β*** is an unknown vector of fixed effects; ***Z*** is the known incidence matrix of random effects, relating the observations in *Y* to ***u***; ***u*** is an unknown vector of random effects, with a multivariate normal (MVN) distribution with mean vector 0 and a variance-covariance matrix denoted by ***G***, that is, ***u*** ~ *N*(**0**, ***G***); and ∈ is an unknown vector of random residuals with an MVN distribution with mean vector 0 and a positive-definite variance-covariance matrix denoted by ***R***, that is, ∈ ~ *N*(**0**, ***R***). Both ***u*** and ∈ are independent. In our experiment, the genetic variance component (*δ*^2^*g*) consists of the F_2:3_ hybrid genotype effect, the interaction between the genotype and block effect, and the replication nested within a block effect, in which all of these components, including the residual variance (*δ*^2^∈), were treated as random effects, whereas the overall mean was treated as fixed effect. The analyses for mixed models were performed using ASReml-R 3 (Butler et al. 2009).

**Fig. 2:**
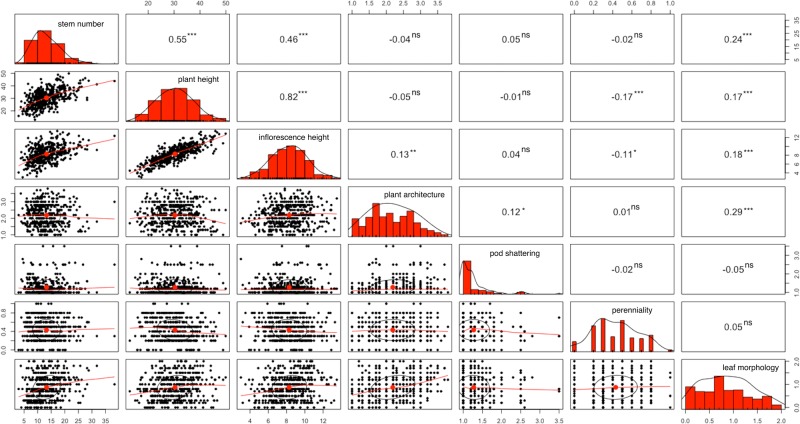
Correlations (*r*) (above diagonal) and scatter plot matrices (below diagonal) among domestication traits of field cress. Diagonal plots represent histogram distributions for domestication traits. The ns indicates the *r* value is statistically non-significant. The number of asterisk(s) show the level of significance (*P < 0.05), (**P < 0.01) and (***P < 0.001).

Broad sense heritability (*H*^2^), estimated (Table 2) as a ratio of genetic variance to the phenotypic variance (Falconer and Mackay 1996), includes both genetic and residual variance components. The correlations (**r**) between domestication traits (Fig. 2) were computed as given by Steel and Torrie (1980). Significance of each correlation was determined using a *t*-statistic test, after a *z*-score transformation of the correlation coefficient, to examine the hypothesis stating that the correlation is different from zero (i.e., different from nolinear association). We examined the coefficient of determination to estimate the amount of shared variation between traits with the help of ggm library (Marchetti 2006). Correlations between domestication traits were also implemented in R-3.6.1 (R Core Team 2018).

### DNA Isolation and genotyping

DNA was isolated from young and fresh field cress leaves of the F_2_ progenies using cetyl trimethylammonium bromide (CTAB) protocol with some modifications in Saghai-Maroof et al. (1984) method. SNPs were developed from restricted amplified DNA (RAD) sequences sampled from the accessions of *Lepidium* (Lopes-Pinto et al. 2016). DNA samples of the 428 F_2_individuals were sent to Edinburgh Genomics (http://genomics.ed.ac.uk) for genotyping using iSelect Illumina Infinium technology. The genotype calls and image files were processed using GenomeStudio (Illumina Inc. San Diego, CA, USA) to extract the data. Problematic SNP markers were identified by their poor cluster-separation values—that is lower Norm R and Norm Theta scores. These poor-quality SNPs were visually assessed and excluded, and 7624 SNPs were finally employed to genotype the 428 *F*_2_ individuals of *L*. *campestre*.

### Genetic linkage map construction

This analysis included marker loci and individuals with less than 5% missing values. After pre- and post-map quality control, 1461 of the 7624 SNP markers, in 428 individuals, were used for the final linkage map construction of field cress. Genetic map construction was carried out with JoinMap 4.1 (Van Ooijen 2006) using maximum likelihood mapping algorithm. The independent logarithm of the odds (LOD) parameters, ranging from 3 to 11, were used to assign linked markers to their corresponding linkage groups (LGs). A linkage map was constructed for each LG using combinations of Gibbs sampling, simulated annealing, and spatial sampling. Linkage mapping function was computed using Haldane’s mapping function (Haldane 1919), and LG maps were derived through MapChart 2.2 (Voorrips 2002). Synteny analysis data were accessed from previous research on *L*. *campestre* (Desta et al. 2019). Allele frequencies with one of the alleles, p, minus 0.5, were computed to determine the expected allele frequencies for both F_2_ population and candidate QTL.

### QTL map analysis

Phenotypic data were initially checked to see whether they follow a normal distribution, which was determined by the probability (*P*) values of the Shapiro–Wilk test for normality—an assumption of the analysis of variance (ANOVA). According to this test, both plant and inflorescence heights met the criteria of normality.

Phenotypic data of F_2:3_, along with the SNP scores of the mapping population and linkage maps of field cress, were imported and analysed in MapQTL^®^6 (Van Ooijen 2009). We quantified the explained phenotypic variation (*R*^2^) for each trait, as well as the additive variance for plant height, to demonstrate the variation in domestication traits. The relative positions and effects of putative QTL on plant and inflorescence heights were analyzed with initial interval mapping (IM) followed by an automatic backward elimination of cofactors recognized in the IM. The loci detected in backward elimination were fitted again to use the multiple QTL mapping (MQM) method (Jansen and Stam 1994; Zeng 1994). MQM is a robust algorithm as it combines multiple regression analysis with IM (Jansen and Stam 1994) and fits the significant loci in the same or different chromosomes as a cofactor to increase the precision of QTL detection while minimizing the residual variations.

Non-parametric estimator Kruskal–Wallis (KW) rank-sum test was applied to analyze the QTL for leaf morphology, plant architecture, pod shattering, perenniality, and stem number. The KW rank-test, equivalent to a one-way ANOVA, approximately follows the chi-square distribution (*χ*^2^), including its degree of freedom, which assumes, under null hypothesis, that there is no effect on the segregating QTL (Van Ooijen 2009). KW test was initially employed with *P* < 0.005 significant threshold level to examine the QTL-marker associations. This was followed by a further analysis of the significant loci from the KW test using MQM approach to locate and estimate the QTL effects.

LOD scores surpassing a genome-wide error rate of 5% (*P* ≤ 0.05), using 10,000 permutations (Churchill and Doerge 1994), were considered a significant association between SNP and trait loci. The mapping step size was 1 cM with a maximum of 5 neighboring markers and 200 iterations. The support interval was established as a 95% support level or confident interval (CI) for two corresponding LOD drop-offs on either sides of the surrounding QTL peak. QTL with an overlapping support level were represented by the highest peak value in that CI. To ascertain the epistasis effects across multiple QTL candidates, we studied the interaction effect among candidate loci in R-3.6.1 (R Core Team 2018).

## Results

### Phenotypic evaluations

#### Correlation and shared variation between traits

The correlation coefficients (*r*) between measured phenotypic traits ranged from 0.01 to 0.82 (Fig. 2). Plant height had a statistically significant positive correlation (*r* = 0.82; *P* < 0.001) with inflorescence height (Fig. 2). Similarly, significant positive correlations were noticed, for example, between plant height and stem number (*r* = 0.55; *P* < 0.001) and stem number and leaf morphology (*r* = 0.24, *P* < 0.001). The correlations of perenniality to all other traits were insignificant except for plant height (*r* = −0.17; *P* < 0.001) and inflorescence height (*r* = −0.11, *P* < 0.05; Fig. 2). We only found positively significant correlation (*r* = 0.12; *P* < 0.05) between pod shattering and plant architecture, whereas the remaining five traits revealed insignificant correlations with pod shattering.

The shared variations between traits were examined with (above diagonal; Table 1) and without keeping (below diagonal; Table 1) five of the seven domestication traits constant. A comparison of the variations showed that those contributing to below diagonal, for stem number versus plant height (30.1%) and for stem number versus inflorescence height (21.1%), were substantially higher than their shared variations above diagonal (9.7 and 0.1%, respectively). These huge increments can be attributed to the additional variations from the remaining five domestication traits below diagonal (Table 1). On the other hand, in some traits, for instance, contribution of variation between plant architecture and inflorescence height below diagonal (1.7%) was smaller than above diagonal (8.2%), possibly due to the antagonistic contributions of shared variations outweighing the positive contributions.

**Table 1:** The proportion of variation (%) shared between phenotypic traits of *Lepidium campestre*.

Traits	Arch.^a^	Stem nr	Plant height	Infl. ht.^b^	Pod sht.^c^	Pern.^d^	Lf mor.^e^
Arch.^a^	–	0.8	6.4	8.2	1.6	0.1	10.4
Stem nr	0.2	–	9.7	0.1	0.7	0.5	3.8
Plant height	0.3	30.1	–	60.1	0.4	2.9	0.6
Infl. ht.^b^	1.7	21.1	66.6	–	0.3	0.4	0.2
Pod sht.^c^	1.4	0.2	0.0	0.2	–	0.1	1.0
Pern.^d^	0.0	0.0	2.9	1.1	0.0	–	0.5
Leaf mor.^e^	8.6	5.9	3.0	3.1	0.3	0.3	–

The variation accounted between phenotypic traits while all other five traits held constant. The variance contribution below diagonal indicates without accounting for the shared variations from the remaining five traits.

^a^Plant architecture.

^b^Inflorescence height.

^c^Pod shattering.

^d^Perenniality.

^e^Leaf morphology.

#### Verification for pod shattering in field cress

**Fig. 3:**
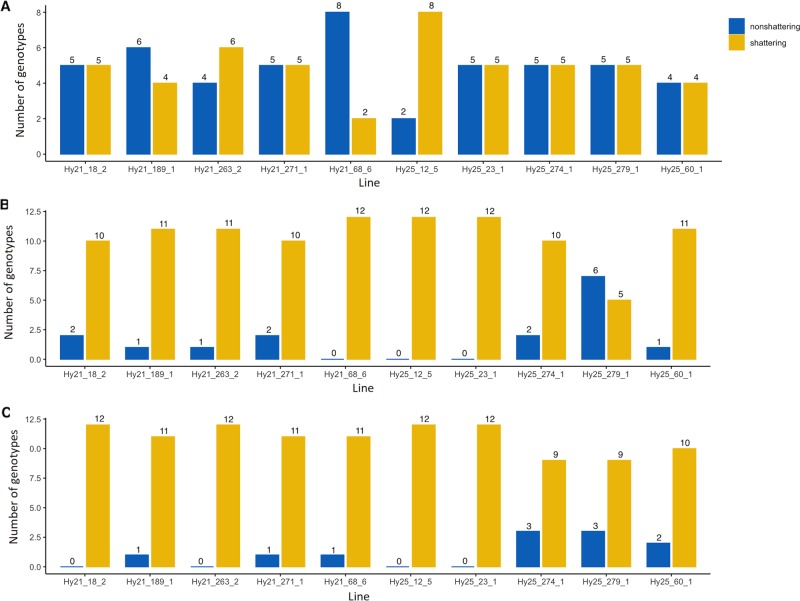
Validation of the non-shattering lines of F_2:3_ population across years and sites. **a** The F_4_-generation with 10 replications in 2015 at one site that is, Alnarp, southern Sweden. **b** The F_5_-generation with 12 replications in 2016 at Alnarp. **c** The F_5_-generation with 12 replications in 2016 at Uppsala (central Sweden).

To test the performance of pod shattering, specifically its potential to increase the yield of field cress, we chose from the best performing non-shattering individuals of F_2:3_ families, and further phenotyped these advanced progenies across years and sites. Shattering genotypes, obscured in non-shattering F_2:3_ population, were revealed in the segregating individuals of F_4_ lines (Fig. 3a). In the F_5_ lines, non-shattering genotypes declined a lot and even became nil in some lines (e.g. Hy25_12_5 and Hy25_23_1 lines at both sites; Fig. 3b, c). In subsequent generations, non-shattering genotypes declined dramatically whereas sensitive genotypes for pod shattering substantially increased (Fig. 3a–c).

### Genetic linkage mapping

After quality control, 1461 of the 7624 SNP loci were mapped to eight LGs (Supplementary Fig. 1) with a total map length of 571.9 cM (Table 2). LG7 had the largest lenght with 124.9 cM, whereas the highest number of markers were mapped to LG1 (409). Closer inspection of skewed loci incorporating ~30% (440 of the 1461) of the SNP genotypes deviated (*P* < 0.05) from the Mendelian segregation ratios (Table 2). LG6 (173 loci) showed the highest segregation distortions followed by LG7 (86 loci), and then by LG3 (80 loci). No segregation distortion was detected in LG1, LG5, and LG8 (Table 2).

**Table 2:** Number of SNP markers, length of linkage groups (LGs) in cM, number of conserved syntenic and distorted loci in each LG of field cress.

LG	Locus	Size	Synteny	SegSyn^a^	Segregation distortion						
					A	B	C	D	E	F	Total
1	409	77.2	260	–	–	–	–	–	–	–	–
2	209	74.2	101	44	21	9	13	–	–	1	44
3	145	59.7	93	43	6	5	15	6	13	35	80
4	180	75.0	73	30	15	1	2	2	10	27	57
5	84	90.9	35	–	–	–	–	–	–	–	–
6	207	46.1	122	99	55	39	32	17	22	8	173
7	168	124.9	100	39	42	8	12	3	12	9	86
8	59	23.9	41	–	–	–	–	–	–	–	–
Total	1461	571.9	825	255	103	44	37	7	22	42	440

^a^Segregation distortion and synteny, the level of distorted SNP loci is indicated by A = *P* < 0.05, B = *P* < 0.01, C = *P* < 0.005, D = *P* < 0.001, E = *P* < 0.0005 and F = *P* < 0.0001.

Across the eight LGs of *L*. *campestre*, ~57% (825 of 1461) of the SNP sequences were in conserved synteny while relating with *A*. *thaliana* chromosomes (Table 2). The highest and the lowest conserved synteny were identified in LG1 (260 loci) and LG5 (35 loci), respectively. Considering segregation distortion and homologies of loci, 255 of the 825 conserved synteny loci (~31% of the conserved synteny loci or ~17% of the SNP loci) showed pairing of both skewed segregation and conserved synteny (Table 2). Similar to the segregation distortion alone, LG6 showed the highest record (99 loci) when segregation distortions and conserved synteny loci were combined.

### QTL map analyses

The most common segregations of favorable QTL alleles from field cress parents include increased plant and inflorescence heights, upright and semi-upright plant architectures, vigorous stem growth, and indehiscence (relative to *L*. *heterophyllum*). On the other hand, perenniality, semi-prostrate and prostrate plant architectures, as well as highly indented or lobed leaf morphology (relative to field cress) are the possible favourable QTL segregations from the *L*. *heterophyllum* parent.

**Fig. 4:**
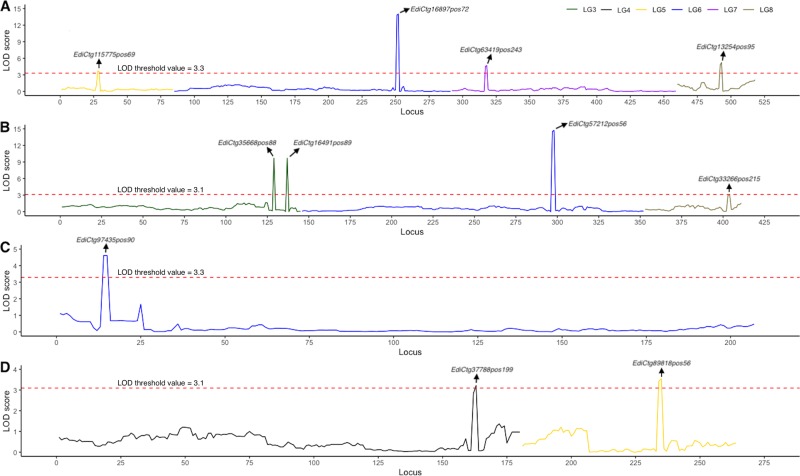
The detection and estimation of QTL. **a**, **b** Plant and inflorescence heights. **c** Stem number. **d** Perenniality. The LOD scores are shown on the y-axis and the loci id numbers are ordered based on the linkage map position. Arrows are used to indicate candidate loci for domestication traits. The red dashed lines are used to indicate the LOD threshold values.

**Fig. 5:**
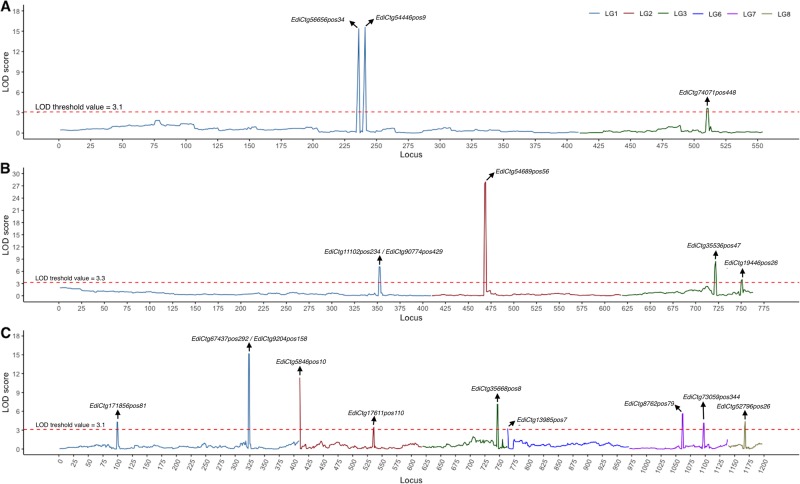
The detection and estimation of QTL. **a**–**c** For pod shattering, plant architecture and leaf morphology, respectively. The LOD scores are shown on the y-axis and the loci id numbers are ordered based on the linkage map position. Arrows are used to indicate candidate loci for domestication traits. The red dashed lines are used to indicate the LOD threshold values.

**Fig. 6:**
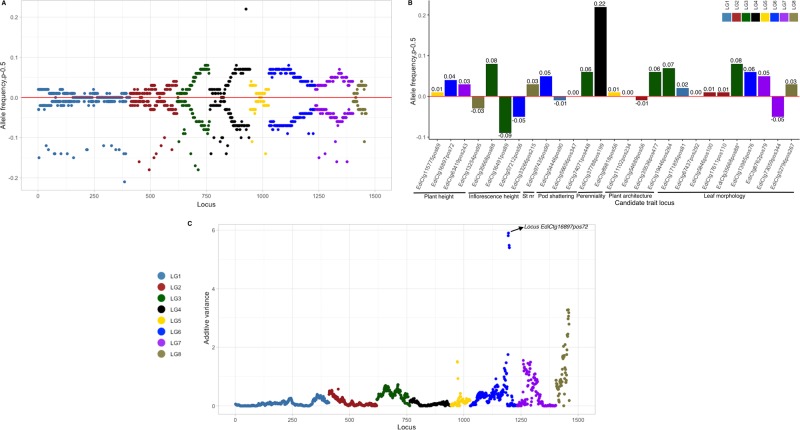
Allele frequency with one of the alleles, p, −0.5 in both F2 population and QTL candidates, and the additive variance of plant height. **a**, **b** The frequency of p−0.5 at each locus in F_2_ population, as well as at each candidate trait locus. Values above and below the abscissa represents positive and negative values, respectively. The patterns of the expected allele frequencies of the F_2_ and candidate QTL were visualised compared with the expected allele at abscissa. The red line at the abscissa indicates the expected frequency of allele. The domestication trait names are indicated below the dashed lines and the st nr is abbreviated for stem number. **c** The additive variance of plant height represented at each locus. The arrow indicating the candidate locus associated with the highest peak of additive variance at linkage group 6 (LG6) and this locus also explained 12.4% of the total phenotypic variation in plant height (Table 3). The loci id numbers along the x-axis in **a**, **c** are ordered based on the linkage map position.

We identified a total of 27 QTL effects in seven domestication traits of field cress using MQM algorithms (Fig. 4a–d; Fig. 5a-c; Table 3). The support interval size varied between 0–0.1 and 13.3–16.7 cM. The proportion of total explained phenotypic variation by QTL (*R*^2^) ranged from 4.9% (for stem number) to 39.8% (for leaf morphology). Consistent with the explained phenotypic variation in plant height (*R*^2^ = 12.4%; Table 3), the highest peak in additive variance for plant height was obtained for LG6 (Fig. 6c). Small effects of QTL were identified across all LGs with all domestication traits, while LG1 (for both leaf morphology and pod shattering), LG2 (for plant architecture), and LG6 (for both plant and inflorescence heights) showed the major effect QTL regions in the field cress genomes.

**Table 3:** Quantitative trait loci (QTL) for seven domestication traits.

Trait	Locus	LG	LOD	Position (cM)	Confidence interval (cM)	*R*^2^ (%)	Total *R*^2^	*H*^2^
Plant height	*EdiCtg115775pos69*	5	3.7	66.2	66.2–67.2	3.1	23.8	47.9
	*EdiCtg16897pos72*	6	14.0	39.7	39.1–39.8	12.4		
	*EdiCtg63419pos243*	7	4.7	3.3	2.6–3.5	3.9		
	*EdiCtg13254pos95*	8	5.3	17.2	16.8–17.3	4.4		
Inflorescence height	*EdiCtg35668pos88*^*a*^	3	9.7	49.7	49.7–50.2	8.1	26.3	53.7
	*EdiCtg16491pos89*	3	9.7	52.0	51.9–52.1	3.1		
	*EdiCtg57212pos56*	6	14.8	36.6	36.3–36.8	12.6		
	*EdiCtg33266pos215*	8	3.1	22.6	22.2–23.0	2.5		
Stem number	*EdiCtg97435pos90*	6	6.6	3.5	3.5–4.7	4.9	4.9	37.0
Pod shattering	*EdiCtg54446pos90*	1	15.4	41.2	40.7–41.4	14.8	36.8	87.9
	*EdiCtg56656pos347*	1	15.3	42.1	42.0–42.6	15.1		
	*EdiCtg74071pos448*	3	3.6	39.6	39.4–40.3	3.3		
Perenniality	*EdiCtg37788pos199*	4	3.2	71.3	70.7–71.4	3.3	6.9	18.7
	*EdiCtg89818pos56*	5	3.6	81.0	80.2–81.2	3.6		
Plant architecture	*EdiCtg11102pos234/EdiCtg90774pos429*	1	7.1	66.4	66.3–66.5	4.1	29.1	87.0
	*EdiCtg54689pos56*	2	28.0	16.4	15.5–16.6	17.9		
	*EdiCtg35536pos477*	3	8.5	41.2	39.6–42.2	4.9		
	*EdiCtg19446pos264*	3	4.0	50.9	50.2–51.0	2.2		
Leaf morphology	*EdiCtg171856pos81*	1	4.3	15.0	14.9–15.2	2.8	39.8	69.2
	*EdiCtg67437pos292/EdiCtg9204pos158*	1	15.2	61.0	60.8–61.1	10.7		
	*EdiCtg5846pos100*	2	11.4	0.0	0.0–0.1	7.8		
	*EdiCtg17611pos110*	2	3.5	48.3	47.4–48.4	2.3		
	EdiCtg35668pos88^*^	3	7.1	49.7	49.6–50.2	4.8		
	*EdiCtg13985pos76*	6	3.3	0.0	0.0–0.8	2.1		
	*EdiCtg8762pos79*	7	5.6	13.4	12.8–13.5	3.7		
	*EdiCtg73059pos344*	7	4.1	105.3	104.1–106.3	2.7		
	*EdiCtg52796pos267*	8	4.4	16.1	13.3–16.7	2.9		

It represents the location as described with each linkage group (LG), LOD score value, the proportion of phenotypic variance explained (*R*^2^, %) by individual (each candidate locus) and joint QTL (total *R*^2^ per trait), as well as the broad sense heritability (*H*^2^) of each trait.

^a^A locus expressed in both inflorescence height and leaf morphology.

Compared with *Arabidopsis* genome, 13 of the 27 field cress candidate QTL had conserved synteny (Supplementary Data S1), four of which had skewed segregations in addition to synteny. To investigate the distributions of the expected allele frequencies, we computed and visualized it in both F_2_ population (Fig. 6a) and candidate QTL (Fig. 6b). Except for one candidate, QTL associated with perenniality (0.22; Fig. 6b), the pattern was relatively either close or at the expected allele frequency.

Multiple QTL were detected for all domestication traits except in stem number. To unravel the epistasis effects, we analyzed the interaction effects among candidate QTL. Based on this analysis, we found significant (*P* < 0.001) interaction effects among candidates of pod shattering QTL (Supplementary Data S2). However, the remaining five domestication traits did not show any significant interaction effects among the candidate QTL. The broad-sense heritability (*H*^2^) of traits varied between 18.7% (for perenniality) and 87.9% (for pod shattering) (Table 3).

## Discussion

A total of 27 QTL were estimated across all LGs in seven domestication traits (Fig. 4a–d; Fig. 5a–c; Table 3), 13 of which included significantly (*P* < 0.05) skewed segregations (Supplementary Data S1). In linkage analysis, meticulous incorporation of skewed loci could have numerous biological benefits, such as to (i) attain good resolution of genetic maps (Zuo et al. 2019); (ii) find candidate QTL that are associated with traits of interest (Xu 2008); and (iii) gain access to conserved synteny compared with a reference genome (Table 2). In this study, excluding the distorted loci could have led to losses not only in 255 conserved synteny (Table 2), but also in 13 of the 27 candidate QTL (Supplementary Data S1).

Six major effect QTL (for pod shattering, leaf morphology, and plant inflorescence heights) were identified across three LGs (LG1, LG2, and LG6) of *L*. *campetsre* (Fig. 4a, b; Fig. 5a–c; Table 3). Large effect QTL are often stable across multiple environments (Dixit et al. 2014; Kato et al. 2014), and practically feasible for map-based or positional cloning of underlying genes (Mackay 2009). A major effect locus study was used to effectively position the candidate gene underlying the plant height QTL in maize (Teng et al. 2013). Thus, major effect QTL identified in this study may be key to positional or functional cloning of candidate genes in field cress improvement.

Plant architecture and plant height are interrelated traits that affect the yield of plants. Tall rapeseed plants, for example, are found to be prone to lodging, thereby affecting the seed yield at harvest (Mei et al. 2009). Plant architecture is a key feature that distinguishes a biennial field cress from a perennial *L. heterophyllum*. Erect and semi-erect stem positions are the usual features of field cress, while prostrate or semi-prostrate positions (see material and methods sections for their descriptions) are the typical plant architectures of *L. heterophyllum*. Stem lodging is one of the major challenges that a domesticated field cress must overcome.

Characterization of candidate domestication genes responsible for plant architecture was reported in crop species. The *teosinte branch 1* (*tb1*) that represses lateral branching in maize (Doebley et al. 1995, 1997) also has a pleiotropic effect on various plant architecture-related traits (Clark et al. 2006). The deactivation of the *PROSTRATE GROWTH 1* (*PROG1*) gene that leads the prostrate growth toward erect position improves yield in rice (Jin et al. 2008; Tan et al. 2008). The identification of deletion genes associated with *Rice Plant Architecture Domestication* (*RPAD*) locus in both African and Asian rice indicates a transfer site of the prostrate resulting in changing the low-yield wild rice to erect and high-yield domesticated rice (Wu et al. 2018). In addition, inactivating the large effect QTL *Tiller Angle Control 1* (*TAC1*) transfers the non-compact architecture to compact and erect tillers in rice (Yu et al. 2007). Together, these results indicate that genes that regulate the QTL of plant architecture-related traits can not only be used to transfer the prostrate to erect position, but can also improve the potential yield of plants.

Pod dehiscence is another important domestication trait that affects the yield of several plant species (Funatsuki et al. 2014; Gan et al. 2008; Jie et al. 2017; Mohammed et al. 2014). Although field cress and *L. heterophyllum* both undergo severe pod shattering, the former has relatively lower shattering than the latter. A loss of ~0.5 t ha^−1^ is reported due to pod shattering in canola (*Brassica* *juncea*) unless early harvest is practised (Gan et al. 2016). With the identification of double mutant *Shatterproof-1* and *Shatterproof-2* genes, indehiscent pods were obtained in *A*. *thaliana* (Liljegren et al. 2000). Similar results of reduced shattering have been reported in rice (Konishi et al. 2006; Li et al. 2006; Lin et al. 2007; Wu et al. 2017), and soybean (Dong et al. 2014; Funatsuki et al. 2014). Collectively, these results suggest that finding a reliable non-shattering QTL followed by isolation and characterization of underlying genes will substantially accelerate the domestication and yield of novel plants—such as field cress.

In dicot species (e.g. members of Brassicaceae family), formation of the abscission layer is introduced to induce pod shattering near or after maturity (Dong and Wang 2015; Patterson 2001). Research in *Arabidopsis* revealed that comprehensive regulatory networks (e.g. the MADS box transcription factor) and tissues (Kay et al. 2013; Liljegren et al. 2000; Liljegren et al. 2004), as well as phytohormones (Arnaud et al. 2010; Cecchetti et al. 2008; Marsch-Martínez et al. 2012; van Gelderen et al. 2016) are the hallmarks to regulate the process of pod shattering. Given the close phylogenetic position for both field cress and *Arabidopsis*, coupled with the glimpse of high-throughput sequencing, the above results can be translated into field cress to understand the physiological and molecular trajectories responsible for pod shattering.

Our validation of phenotyping across environments and years demonstrated that as the number of generations increased, the number of non-shattering individuals decreased drastically, and the shattering genotypes sharply increased (Fig. 3a–c). The decline of non-shattering genotypes across generation was probably driven by the phenomenon of inbreeding followed by selection (Bosse et al. 2019; Jamieson et al. 2008). Inbreeding is, however, unavoidable biological event, which could be used to generate pure lines as well as to increase the genetic similarity among relatives (Walsh and Lynch 2018). Genetic drift may also eliminate favourable alleles while fixing the disadvantageous alleles (Gaut et al. 2018; Wright et al. 2008). Thus, simultaneous inbreeding and selection in a small effective population size may also dramatically eliminate the useful mutation from a population.

Considering the agro-ecosystem, perennial plants have manifold benefits, such as eliminating the need for soil tillage, efficient utilization of natural resources (King and Blesh 2018), improving soil-carbon composition, and minimizing soil degradation (DeHaan and Van Tassel 2014). On the other hand, perenniality also impedes agricultural tillage and harvesting machines when intercropped with annual cereals. Furthermore, intercropping perennially developed *L. campestre* (as a consequence of crossbreeding with *L.* *heterophyllum*) in arable lands could have a reverse effect on soil nitrogen, accelerating nutrient leaching that leads to environmental contamination such as nitrogen pollution (Desta et al. 2019; Kanter et al. 2019). Field cress can minimize nitrogen or phosphorous leaching if it is planted in spring (Ulén and Aronsson 2018). However, the authors also indicated that nutrient leaching could be intensified due to over-mineralization if field cress is inter-planted in autumn. Hence, in crop husbandry, caution is warranted to interpret not only the influences with perenniality but also the inter-sowing season of field cress.

Similar to other weed species (Ikram et al. 2014), field cress also has a severe problem of seed dormancy. We have noticed that some accessions of field cress can remain without germination for an extended period in the soil unless seeds are treated with chemicals—such as gibberellic acid (GA). Recent research in soybean (Jang et al. 2015; Sun et al. 2015; Wang et al. 2018), rice (Sugimoto et al. 2010), *Medicago truncatula* (Chai et al. 2016), and barley (Sato et al. 2016) sheds light on mechanisms of regulating seed dormancy in plants. In addition to finding the naturally existing ecotypes, further efforts are needed in using mutation-based screening for reduced dormancy to investigate seed dormancy in field cress.

The environment is one of the vital components that determines the growth and development of plants. To meet the temperature requirement, we vernalised seedlings of field cress before transplanting them in the field; however, we noticed some variability in flowering. Hence, further research on photoperiod regulation and transition to flower initiation, as well as genes that control vernalisation, is central to facilitate field cress domestication.

In conclusion, to the best of our knowledge, this is the first study focusing on the estimation of QTL effects on seven agro-morphological traits that are key to facilitate field cress domestication. The correlation among these traits may not only assist in selection, but also contribute to examine pleiotropy effects when multiple QTL are involved. We found relatively small support sizes (ranging 0.1–3.4 cM) in the analyzed QTL regions, mimicking fine mapping in which SNPs are interlinked with each other to precisely locate the QTL positions. We found six large effect QTL, whereas the remaining 21 candidate QTL showed small effect QTL. Majority of the identified QTL captured moderate to high heritability, roughly >40%, demonstrating that genomics-assisted selection could deliver improved lines within a few generations of field cress breeding. The use of high-throughput genotyping platform (e.g. SNPs discovered from high-throughput sequencing) can immensely accelerate the process of domestication in novel crop species. The QTL results presented here may eventually provide avenue for map-based or mutation-based (e.g. CRISPR-induced mutations) QTL cloning; introgression of favourable genes in recipient parents; and genomics-assisted breeding approaches (e.g. genomic selection) in field cress domestication.

## Perspective

While the identified QTL in this study could provide valuable contributions in QTL cloning and genomic-assisted breeding, future efforts are remarkably important in getting complete reference genome of field cress. This refence sequence will broaden and advance the in-depth understanding not only on how to explore the existing variation, but also on expanding alternatives of genomic tools in field cress domestication and improvement. Equally important, the QTL hotspot regions combined with fully annotated genome sequences are plausible to systematically correlate and answer the complicated questions in domestication genomics.

### Data archiving

Genotype and phenotype data available from the Dryad Digital Repository: 10.5061/dryad.1jwstqjr4 (and via the direct link https://datadryad.org/stash/share/qtSyvROqbnr0aKOGZ8KpRbzpiUAprOpNkzizZkI47Qc).

## Supplementary information

Supplementary Fig. 1 Genetic linkage map for field cress

Supplementary Data S1. Putative QTL of field cress associated with segregation distortion and conserved synteny

Supplementary Data S2. Epistasis effects among candidtate loci of pod shattering
