# Supplementary material for: Molecular mapping and identification of quantitative trait loci for domestication traits in the field cress (Lepidium campestre L.) genome
Source: Heredity (Edinb). 2020 Feb 19;124(4):579–91. doi: 10.1038/s41437-020-0296-x (PMC7080786; doi:10.1038/s41437-020-0296-x)

Supplementary Fig. 1 Genetic linkage map for field cress

Linkage group 1

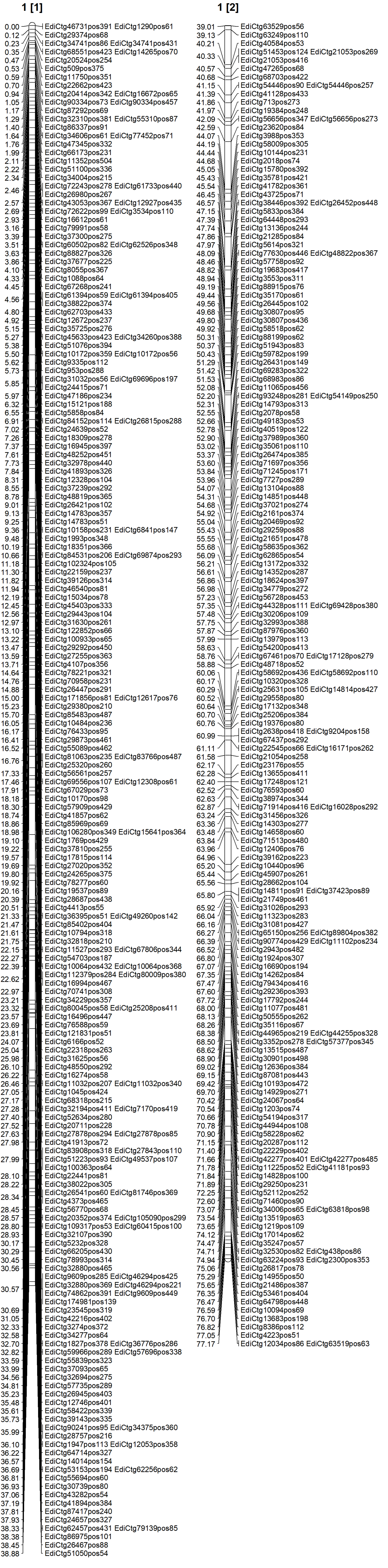

Linkage group 2

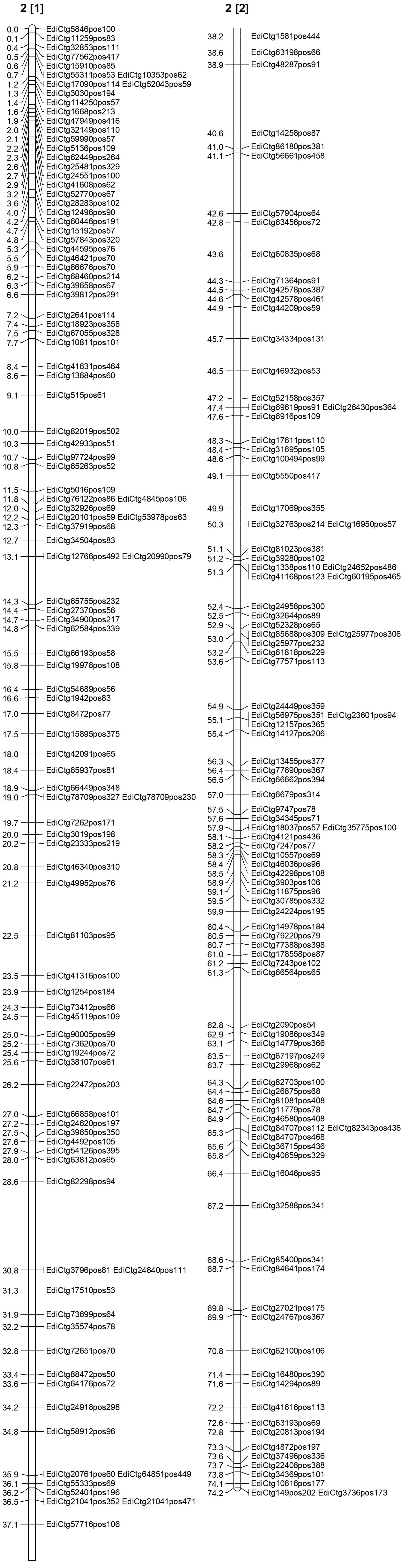

Linkage group 3

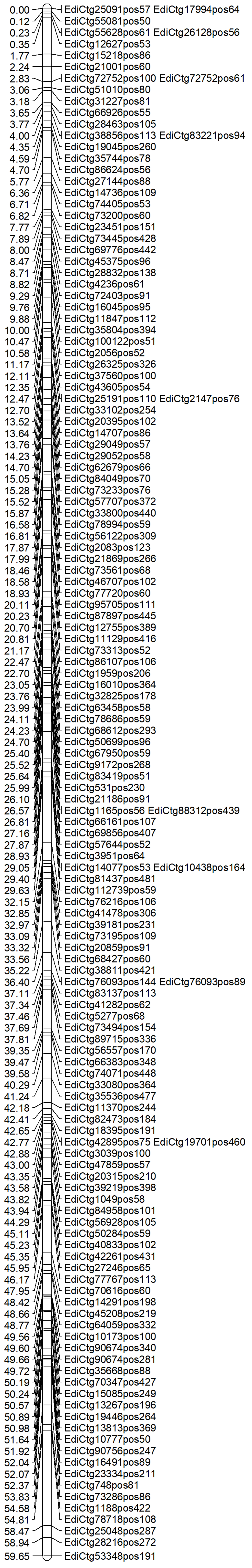

Linkage group 4

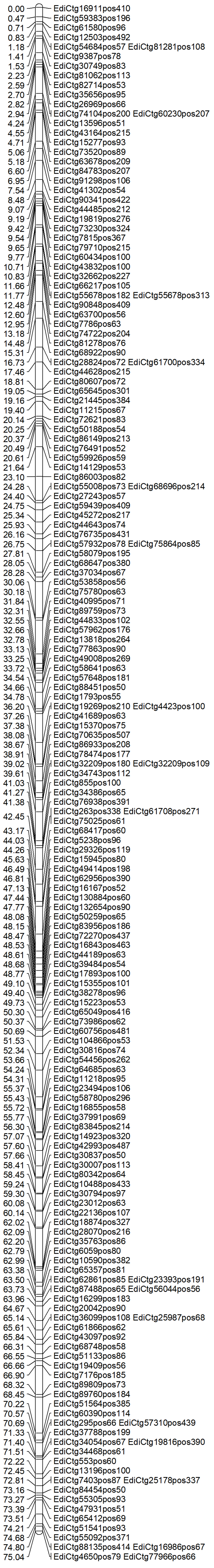

Linkage group 5

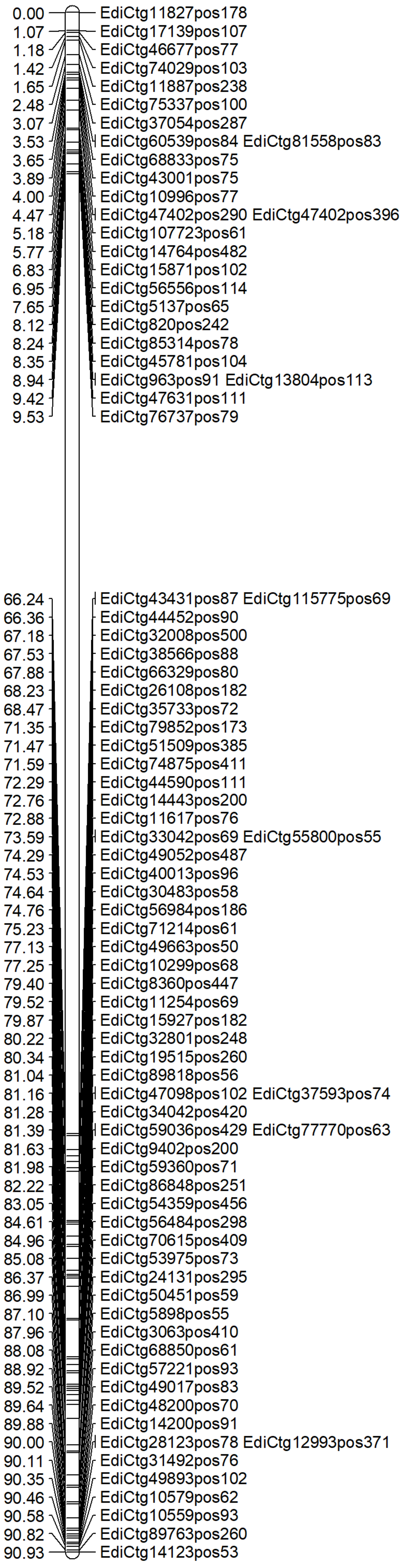

Linkage group 6

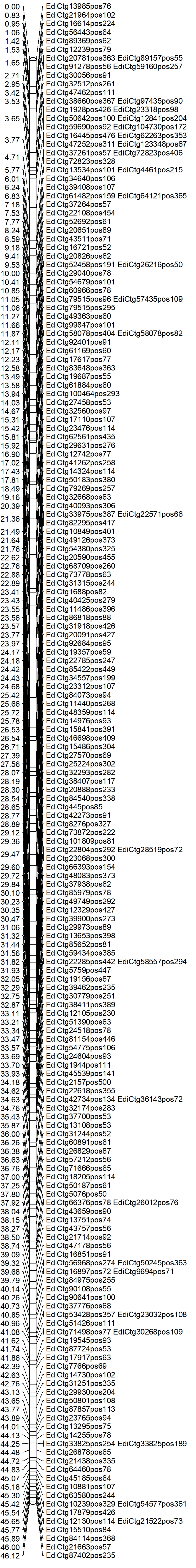

Linkage group 7

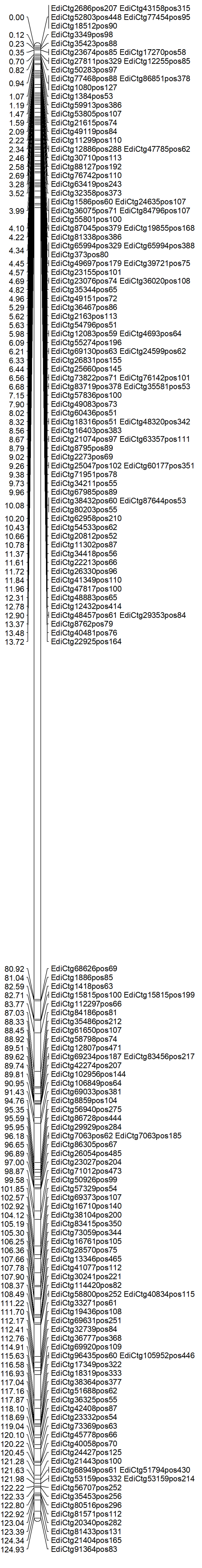

Linkage group 8

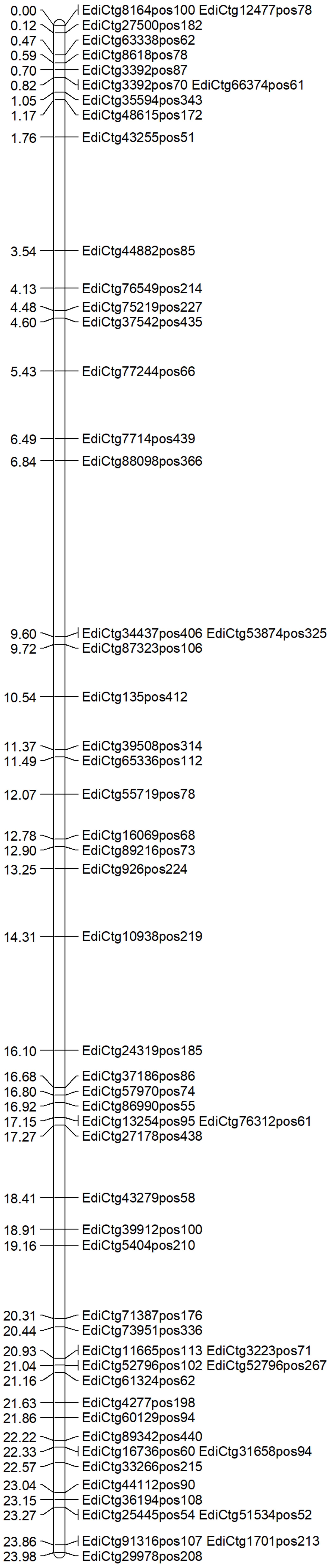

Supplement: Supplementary file 1 — Supplementary Fig. 1 Genetic linkage map for field cress [file 41437_2020_296_MOESM1_ESM.pdf]
