# Supplementary material for: Molecular mapping and identification of quantitative trait loci for domestication traits in the field cress (Lepidium campestre L.) genome
Source: Heredity (Edinb). 2020 Feb 19;124(4):579–91. doi: 10.1038/s41437-020-0296-x (PMC7080786; doi:10.1038/s41437-020-0296-x)
Supplement: Supplementary file 2 — Supplementary Data S1. Putative QTL of field cress associated with segregation distortion and conserved synteny [file 41437_2020_296_MOESM2_ESM.pdf]

Supplementary Data S1. Putative QTL of field cress associated with segregation distortion and conserved synteny

**Trait**	**Locus**	**Linkage group**	**Segregation Distortion P-value**	**Conserved Synteny**
Plant height	*EdiCtg115775pos69*	5	$P \geq 0.05$	Not
	EdiCtg16897pos72	6	P<0.05	Yes
	EdiCtg63419pos243	7	$P \geq 0.05$	Yes
	EdiCtg13254pos95	8	$P \geq 0.05$	Not
Inflorescene height	*EdiCtg35668pos88* [a]	3	$P < 0.0001$	Not
	EdiCtg16491pos89	3	$P < 0.005$	Not
	EdiCtg57212pos56	6	$P < 0.005$	Yes
	EdiCtg33266pos215	8	$P \geq 0.05$	Yes
Stem number	*EdiCtg97435pos90*	6	$P < 0.01$	Not
Pod shattering	*EdiCtg54446pos90*	1	$P \geq 0.05$	Yes
	EdiCtg56656pos347	1	$P \geq 0.05$	Yes
	EdiCtg74071pos448	3	$P < 0.05$	Yes
Perenniality	*EdiCtg37788pos199*	4	$P < 0.005$	Not
	EdiCtg89818pos56	5	$P \geq 0.05$	Yes
Plant architecture	*EdiCtg11102pos234 / EdiCtg90774pos429*	1	$P \geq 0.05$	Yes
	EdiCtg54689pos56	2	$P \geq 0.05$	Not
	EdiCtg35536pos477	3	$P < 0.0001$	Not
	EdiCtg19446pos264	3	$P < 0.0001$	Not
Leaf morphology	*EdiCtg171856pos81*	1	$P \geq 0.05$	Yes
	EdiCtg67437pos292 / EdiCtg9204pos158	1	$P \geq 0.05$	Not
	EdiCtg5846pos100	2	$P \geq 0.05$	Not
	EdiCtg17611pos110	2	$P \geq 0.05$	Yes
	EdiCtg35668pos88	3	$P < 0.0001$	Not
	EdiCtg13985pos76	6	$P < 0.0001$	Not
	EdiCtg8762pos79	7	$P < 0.01$	Yes
	EdiCtg73059pos344	7	$P < 0.05$	Not
	EdiCtg52796pos267	8	$P \geq 0.05$	Yes

a A locus expressed in both inflorescence height and leaf morphology
