# Supplementary material for: Molecular mapping and identification of quantitative trait loci for domestication traits in the field cress (Lepidium campestre L.) genome
Source: Heredity (Edinb). 2020 Feb 19;124(4):579–91. doi: 10.1038/s41437-020-0296-x (PMC7080786; doi:10.1038/s41437-020-0296-x)
Supplement: Supplementary file 3 — Supplementary Data S2. Epistasis effects among candidtate loci of pod shattering [file 41437_2020_296_MOESM3_ESM.pdf]

Supplementary Table 3. Epistasis effect for domestication traits of field cress

Trait	Candidate loci interaction	P-value
Pod shattering	*EdiCtg54446pos90* : *EdiCtg74071pos448*	6.08E-08
	EdiCtg56656pos347 : *EdiCtg74071pos448*	2.49E-07
